# Review of targeted therapy in chronic lymphocytic leukemia: what a radiologist needs to know about CT interpretation

**DOI:** 10.1186/s40644-018-0146-8

**Published:** 2018-04-18

**Authors:** Babina Gosangi, Matthew Davids, Bhanusupriya Somarouthu, Francesco Alessandrino, Angela Giardino, Nikhil Ramaiya, Katherine Krajewski

**Affiliations:** 1 Thoracic Radiology, Brigham and Women’s Hospital, 45 Francis Street, Boston, MA 02115 USA; 2 Harvard Medical School, 25 Shattuck Street, Boston, MA 02115 USA; 3 Chronic Lymphocytic Leukemia, Dana Farber Cancer Institute, 450 Brookline Avenue, Boston, 02284 USA; 4 Radiology, Massachusetts General Hospital, 55 Fruit Street, Boston, MA 02114 USA; 5 Emergency Radiology, Brigham and Women’s Hospital, 45 Francis Street, Boston, MA 02115 USA; 6 Department of Radiology, Dana Farber Cancer Institute, Boston, MA 02284 USA

**Keywords:** Indolent lymphoma, Chronic lymphocytic leukemia, Targeted therapy, Drug related toxicity

## Abstract

The last 5 years have been marked by profound innovation in the targeted treatment of chronic lymphocytic leukemia (CLL) and indolent lymphomas. Using CLL as a case study, we present a timeline and overview of the current treatment landscape for the radiologist, including an overview of clinical and radiological features of CLL, discussion of the targeted agents themselves, and the role of imaging in response and toxicity assessment. The goal is to familiarize the radiologist with multiple Food and Drug Administration (FDA)-approved targeted agents used in this setting and associated adverse events which are commonly observed in this patient population.

## Background

Chronic lymphocytic leukemia (CLL)/ small lymphocytic lymphoma (SLL) is the most prevalent leukemia in older adults in the Western hemisphere, with an estimated 19,000 new cases diagnosed per year in the United States [1]. The treatment landscape of CLL has evolved dramatically in the last 5 years, with the approval of multiple new targeted agents leading to prolonged survival of affected patients. Many of the therapies are also approved by the United States FDA for treatment of other indolent lymphomas, including follicular lymphoma and lymphoplasmacytic lymphoma/ Waldenstrom’s macroglobulinemia [2, 3]. Given the sizable population of patients being treated with these agents and the role of imaging in CLL/ lymphoma management, radiologists should gain familiarity with the drugs and especially, imaging manifestations of drug toxicity.

At present, the spectrum of CLL therapy options includes traditional chemotherapies, multiple antibodies (anti-CD20, anti-CD52) as well as small molecule inhibitors (Bruton tyrosine kinase, phosphatidylinositol 3-kinase, and B cell leukemia/lymphoma-2 inhibitors). In CLL, individualized treatment selection and management decisions are based on the integration of several factors, including genomic assessment, patient co-morbidities and preference, and other factors contributing to prognostication and risk stratification [4]. When patients develop indications for treatment, such as cytopenias, massive or symptomatic splenomegaly or lymphadenopathy, therapeutic agents are generally selected based on patient age, co-morbidities and disease-related biological characteristics [5]. In younger CLL patients with few co- morbidities, chemoimmunotherapy regimens such as fludarabine, cyclophosphamide, and rituximab (FCR) have been standard first-line therapy [6]. In older patients, bendamustine and rituximab (BR), chlorambucil and obinutuzumab, and ibrutinib are all now considered to be reasonable standard first-line therapy options [7]. Other targeted agents have been approved in the relapsed/refractory setting, and several other therapies remain under active investigation.

The magnitude of the economic impact of the available targeted therapies is just starting to manifest [8]. As the targeted agents become more commonly used in both academic and community settings, it is necessary for radiologists to remain abreast of the radical changes in CLL and indolent lymphoma management. In this review, using CLL as a case study, we summarize the clinical and radiological features of this disease and discuss the various targeted therapies used to treat CLL and other indolent lymphomas, with emphasis on the role of imaging in toxicity assessment.

## Chronic Lymphocytic Leukemia (CLL)/ Small Lymphocytic Lymphoma (SLL)

**Fig. 1:**
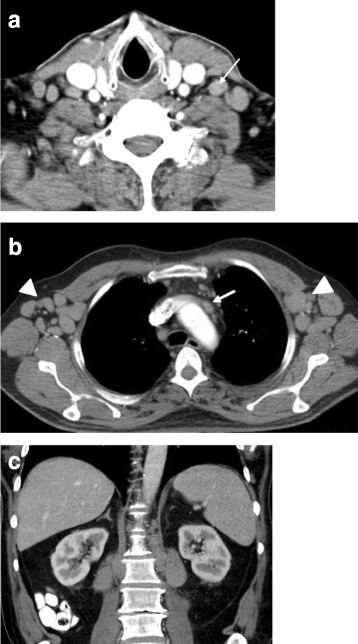
69-year-old woman at the time of diagnosis with CLL. **a** Axial CT image of the neck obtained during arterial phase demonstrates bilaterally enlarged supraclavicular lymph nodes (thin white arrow). **b** Axial CT images of the chest obtained during arterial phase show multiple enlarged bilateral axillary lymph nodes (arrowheads) and mildly prominent prevascular lymph nodes (white arrow). **c** Coronal reconstructed CT image of the abdomen shows mild splenomegaly

CLL and SLL are most common cancers of the elderly, with an average age of presentation of 71 years [5]. CLL and SLL represent a spectrum of the same disease, with CLL manifesting in the peripheral blood and marrow and defined by > 5 × 10 ^9^monoclonal lymphocytes/L, while SLL manifests predominantly in the lymph nodes and spleen, with< 5 × 10^9^monoclonal lymphocytes/L in the blood [4]. The lymph nodes and spleen are involved in both entities, however (Fig. 1), and the prognosis and management of both CLL and SLL is the same.

Patients are commonly referred for evaluation on the basis of peripheral lymphocytosis. Less commonly, patients may present with lymphadenopathy, splenomegaly, frequent infections and/or autoimmune disease. Diagnosis is based on the International Workshop on CLL (iwCLL) 2008 guidelines describing the precise immunophenotype of the blood or marrow lymphocytes, including expression of CD5, CD19, CD20 and usually CD23, among others [5]. Fluorescence in situ hybridization (FISH) is commonly performed, and has important prognostic and predictive power. For example, patients with chromosome 17p deletions and young patients with 11q deletions have been found to have a poorer prognosis as compared to patients with 13q deletion, trisomy 12, or normal FISH [9]. Additional markers with prognostic significance in this disease include presence or absence of somatic mutations of the immunoglobulin heavy chain variable region genes (*IGHV*), expression of the zeta-associated protein 70 (ZAP-70), and somatic mutations of genes such as *TP53*, *NOTCH1*, and *SF3B1* [10, 11]. Older serum markers such as B_2_-microglobulin also have retained prognostic significance.

Staging is clinical and is assessed through physical examination and laboratory studies, using the Rai (US) or Binet (Europe) systems [12, 13]. According to NCCN guidelines, imaging is not necessary, but is commonly performed in advanced stage patients (stage III and IV, characterized by anemia and thrombocytopenia, respectively. A study by Muntanola et al. demonstrated that in Rai stage 0 patients, abdominal disease identified on CT was a predictor of progression, highlighting the possible utility of imaging even in early disease [14]. In this study, abnormal CT was defined by lymph nodes in the abdomen measuring > 10 mm in diameter, multiple clustered nodes in an anatomically defined region measuring < 10 mm in diameter, and/or splenomegaly. In practice, imaging in early stage CLL is generally reserved for patients with high risk disease biology, in particular, those with del (17p) or del (11q), who not infrequently can have bulky abdominal lymphadenopathy out of proportion to their palpable lymphadenopathy on physical examination.

**Fig. 2:**
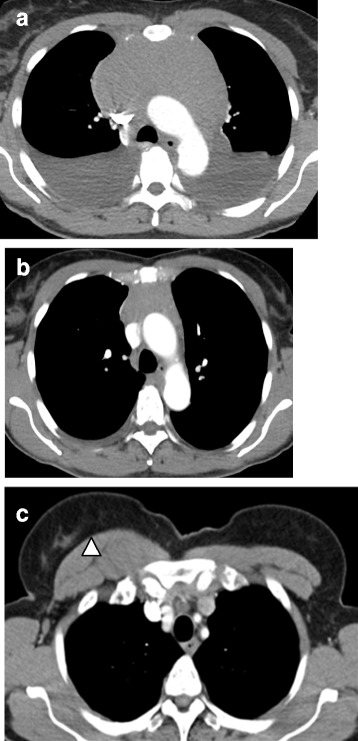
76-year-old woman with chronic lymphocytic leukemia with 11q deletion and unmutated IGHV. **a** Axial CT images of the chest acquired during arterial phase at the time of initial presentation shows a large anterior mediastinal mass measuring up to 10 cm in its largest dimension with bilateral moderate pleural effusions. **b** Axial CT image of the chest obtained during arterial phase 4 months after initiating R-CHOP demonstrates significant decrease in size of the anterior mediastinal mass and improvement of bilateral pleural effusion. **c** Axial CT image of the chest obtained during arterial phase 4 years later demonstrates a new mass in the right sub pectoral region measuring up to 3.5 cm in its largest dimension suspicious for CLL (arrowheads). The lesion was biopsied and was consistent for recurrence of CLL

**Fig. 3:**
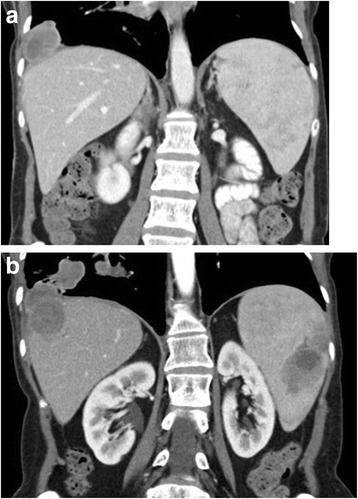
55-year-old woman with CLL treated with rituximab with pain abdomen and fatigue. **a** Coronal reconstructed contrast enhanced CT image of the abdomen and pelvis acquired 4 months before developing new symptoms reveals perihepatic implant with no focal liver or splenic lesions. **b** Coronal reconstructed images of the abdomen and pelvis reveal new focal hypodense lesions in the liver and the spleen with increase in the perihepatic implant and new pulmonary masses. Liver lesion was biopsied and histologic evaluation showed transformation into diffuse large B cell lymphoma

Contrast-enhanced CT (CECT) is the imaging modality of choice in CLL/SLL. Most commonly, CLL/SLL patients demonstrate multi-station mildly to moderately enlarged lymph nodes, with or without splenomegaly or hepatomegaly. Bulky nodes and confluent adenopathy is commonly seen during relapses or, as noted above, in patients at presentation with 17p or 11q deletion (Fig. 2) [15, 16]. Rare sites of CLL involvement have been reported, including the central nervous system [17, 18]. PET-CT is not commonly performed in CLL as it is not often FDG-avid, unless transformation to a higher grade lymphoma (Richter transformation) is suspected, based on the presence of rapidly enlarging lymph nodes, development of “B” symptoms (fevers, drenching night sweats, or unintentional weight loss) or markedly elevated serum lactate dehydrogenase (LDH) (Fig. 3). In these situations, PET-CT is helpful in identifying the most FDG-avid site of disease for tissue sampling and diagnosis [19]. Importantly, SUVmax likely reflects tumor aggressiveness, with or without Richter transformation. In one study of over 300CLL patients imaged with PET-CT, SUVmax ≥10 was a useful discriminator of outcomes, associated with median overall survival 6.9 months compared to patients with SUVmax< 10 and median overall survival 56.7 months [20].

Indications for treatment are defined by the 2008 iwCLL guidelines, including progressive cytopenias, symptomatic or massive splenomegaly or lymphadenopathy, refractory autoimmune disease (usually autoimmune cytopenias), and constitutional symptoms driven by disease progression [5]. It is helpful to perform imaging at baseline prior to the onset of treatment and after the administration of therapy to assess response. Response is also defined according to the 2008 iwCLL guidelines using clinical and imaging criteria. Imaging findings associated with complete response (CR) include no lymph node measuring > 1.5 cm in long axis diameter and no hepatosplenomegaly. Imaging features of partial remission (PR) include a decrease of 50% or more of the sum products of up to 6 nodes, with no increase (> 25%) or newly enlarged node, and/or decreased hepatosplenomegaly by ≥50%. In patients with minimal residual disease (sensitivity of 1 leukemic cell in 10,000 benign lymphocytes), residual splenomegaly after fludarabine, cyclophosphamide, rituximab (FCR) has been shown to have no prognostic significance [21]. A newly-described response category is PR with lymphocytosis (PR-L), which indicates patients who would otherwise meet PR criteria but have a residual lymphocytosis. This situation commonly occurs in patients on B cell receptor (BCR) pathway inhibitors such as ibrutinib or idelalisib who are responding well clinically to therapy and as such it was recognized that the persistent lymphocytosis represents a biological effect of these drugs rather than a sign of resistant disease. Progressive disease is indicated on imaging by the appearance of any new lesion, such as enlarged lymph nodes (> 1.5 cm), splenomegaly, hepatomegaly, or other organ infiltrate, or an increase by 50% or more in greatest determined diameter of any previous site.

### Treatment strategies in CLL/SLL

In the last several years, there has been rapid evolution in the treatment strategies of CLL to a more personalized approach with newly available precision therapy [22]. In the 1990s, CLL was generally treated with alkylating agents like chlorambucil and cyclophosphamide and later the purine analog fludarabine. In the 2000’s, combination regimens of first fludarabine plus cyclophosphamide (FC) and then FC plus the anti-CD20 monoclonal antibody rituximab (FCR) became used more widely. The anti-CD52 monoclonal antibody alemtuzumaband the second generation anti-CD20 monoclonal antibody ofatumumab also came into the clinic and had utility in higher risk and refractory patients. Bendamustine, another alkylating agent, used with or without rituximab, also became another important treatment option at this time. However, despite an excellent initial response to chemoimmunotherapy approaches in most patients, the progression free survival on repeated administration was significantly shorter.

In the last 5 years, targeted novel agents have expanded the available CLL treatment armamentarium dramatically, in particular small molecule inhibitors and newer monoclonal antibodies, providing excellent new treatment options for all CLL patients and particular those who are older and have multiple co-morbidities, poor prognostic features, or relapsed/refractory disease. For example, ibrutinib is now considered the standard of care as first-line therapy for del (17p) CLL [2]. In the following section, we reviewsome of the key monoclonal antibodies and also the recently approved targeted agents employed in the management of CLL, with attention to efficacy and toxicity features relevant to radiologists.

## Rituximab (Anti-CD-20 antibody)

Rituximab is a monoclonal antibody which binds to CD20 expressed on the surface of B cells and causes depletion of malignant B cells primarily through antibody-dependent cellular cytotoxicity and complement-dependent cytotoxicity [23]. It was one of the first targeted drugs approved by the FDA for any cancer, with initial FDA approval for B-cell non Hodgkin lymphomas resistant to other regimens in 1997 [24]. Its label was subsequently broadened in 2006 to be included in combination with cyclophosphamide, vincristine and prednisone (R-CVP regimen), which became the standard of care first line treatment for follicular lymphoma [25]. Rituximab was subsequently approved in 2010 as a first line agent in CLL when given in combination with fludarabine and cyclophosphamide (FCR). Rituximab may be administered along with other agents in both fitter patients (for example, with bendamustine) and in older patients with co-morbid conditions (for example, with chlorambucil) [2]. As in other indolent NHLs, rituximab is sometimes utilized as monotherapy in CLL; however, unlike in those other diseases, response rates and durability of response in CLL are modest, and as such rituximab should not routinely be used as monotherapy in CLL.

On imaging, response to therapy occurs in the form of decreased size of lymph nodes, spleen, liver and other sites of lymphoma. In a clinical trial that led to FDA approval of rituximab (FCR) for CLL, at 3 years after randomization, 65% of patients in the FCR group were free of progression compared with 45% in the FC group [26].

**Fig. 4:**
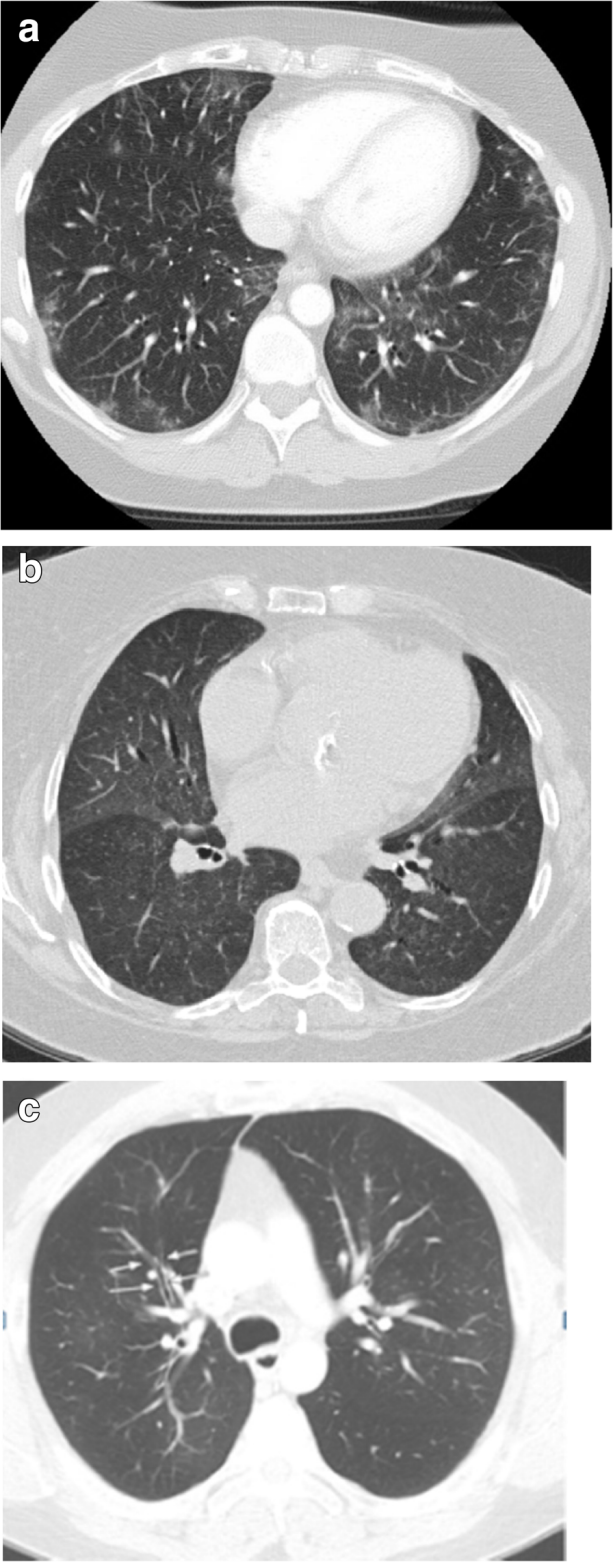
Spectrum of lung toxicities caused by Rituximab. **a** Axial CT images of the chest acquired during arterial phase in a 48-year-old female patient with CLL on maintenance therapy with Rituximab show bilateral ground glass opacities more prominent in the left lower lobe suggestive of drug induced pneumonitis. **b** Axial CT images of the chest in an 84 year old female patient with CLL on Rituximab therapy shows bilateral, symmetric homogenous ground glass opacities in both the lungs consistent with hypersensitivity pneumonitis **c** Axial CT images of the chest obtained during arterial phase in a 45 year old male patient with CLL on maintenance with Rituximab shows hypoattenuated lungs with peribronchial thickening suggestive of bronchiolitis obliterans (thin white arrows)

**Fig. 5:**
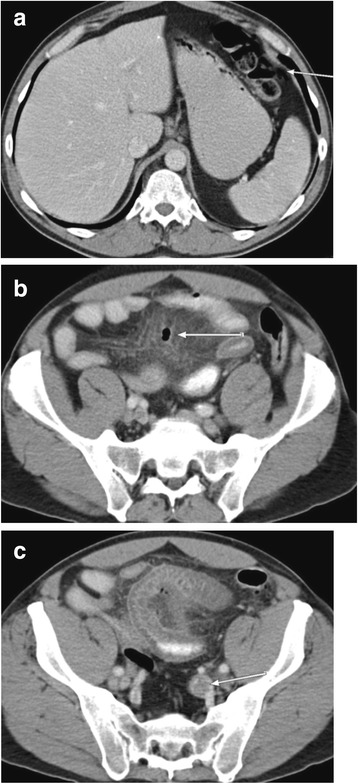
54-year-old man with CLL on maintenance with Rituximab with new onset pain abdomen. **a** Axial CT images of the abdomen obtained during portal venous phase show small focus of air under the left hemidiaphragm (white arrow). **b** Axial CT images of the pelvis obtained during portal venous phase show a focus of air in the mesentery (white arrow) (**c**). Axial CT images of the pelvis obtained during portal venous phase demonstrate thickening of the small bowel loop with a filling defect in the left internal iliac artery (white arrow). Findings were compatible with small bowel perforation

Rituximab toxicities can manifest on imaging (Figs. 4 and 5). Notably, lung toxicities in the form of interstitial pneumonitis and acute respiratory distress syndrome (ARDS) have been well-described, and although they are rare, they can be life threatening [27–29]. Three time-to-onset patterns have been described: ARDS within hours of first infusion, acute/subacute hypoxemic organizing pneumonia within 2 weeks of last infusion, and macronodular organizing pneumonia with insidious/longer onset [27]. On high resolution computed tomography (HRCT), findings include focal or diffuse ground glass opacities and consolidation (Fig. 4) [28]. Rituximab is also known to increase the risk of hepatitis B reactivation in carriers, which can result in acute liver injury, andmanifestson CT with decreased liver attenuation, gallbladder wall edema > 3 mm and mild periportal edema [30]. Progressive multifocal leukoencephalopathy (PML) has also been reported in rituximab-treated CLL patients, which is a rare demyelinating condition caused by JC virus reactivation [31]. On brain MRI, PMLmanifests as FLAIR hyperintense white matter lesions.

## Alemtuzumab (Anti-CD-52 antibody)

Alemtuzumab is an anti-CD52 antibody, directed against the CD52 antigen expressed on the surface of B-cells, T-cells, natural killer cells, eosinophils and macrophages [32]. This antibody acts through complement-mediated and/or antibody-dependent cytotoxicity, and may cause direct apoptosis of B cells [33]. In 2001, alemtuzumab attained initial FDA-approvalunder accelerated approval regulations for B-cell CLL, and full approval was attained in 2007. According to the current NCCN guidelines, alemtuzumab may be employed as a single agent or in combination with rituximab, in the setting of CLL with 17p deletion or relapsed/refractory cases [2].

In the clinical trial that lead to alemtuzumab FDA approval, 297 patients were randomized to either alemtuzumab or chlorambucil. There was a higher overall response rate of 83% in patients treated with alemtuzumab compared to 55% in the chlorambucil arm [34, 35]. Adverse effects in alemtuzumab treated patients included infections, particularly with cytomegalovirus (CMV). The spectrum of severe infectious complications is thought to be related to profound lymphocyte depletion [36]. CMV pneumonitis can be appreciated on CT as bilateral patchy areas of ground glass opacities with centrilobular nodules, consolidation, as well as septal thickening and pleural effusion in some cases [37]. CMV colitis demonstrates a non-specific appearance on CT, associated with wall thickening, edema, mucosal hyperenhancement and perienteric stranding [38]. Additionally, infection with pneumocystis jiroveci pneumonia (PJP), fungal organisms and cerebral toxoplasmosis have been associated with alemtuzumab.

## Ofatumumab (New anti-CD20 antibody)

Ofatumumab is a second generation, type I anti-CD20 antibody with better activation of complement-dependent cytotoxicity and thereby better activity in some cell lines resistant to rituximab and in cells with low CD20 expression [39]. In 2009, ofatumumab was FDA-approved for patients with previously treated CLL. In 2014, ofatumumab was approved in combination with chlorambucil in previously untreated patients, in whom fludarabine is not appropriate [40]. In 2016, ofatumumab was FDA-approved for extended treatment of patients with recurrent or progressive CLL who are in complete or partial response following at least two lines of therapy [41].

**Fig. 6:**
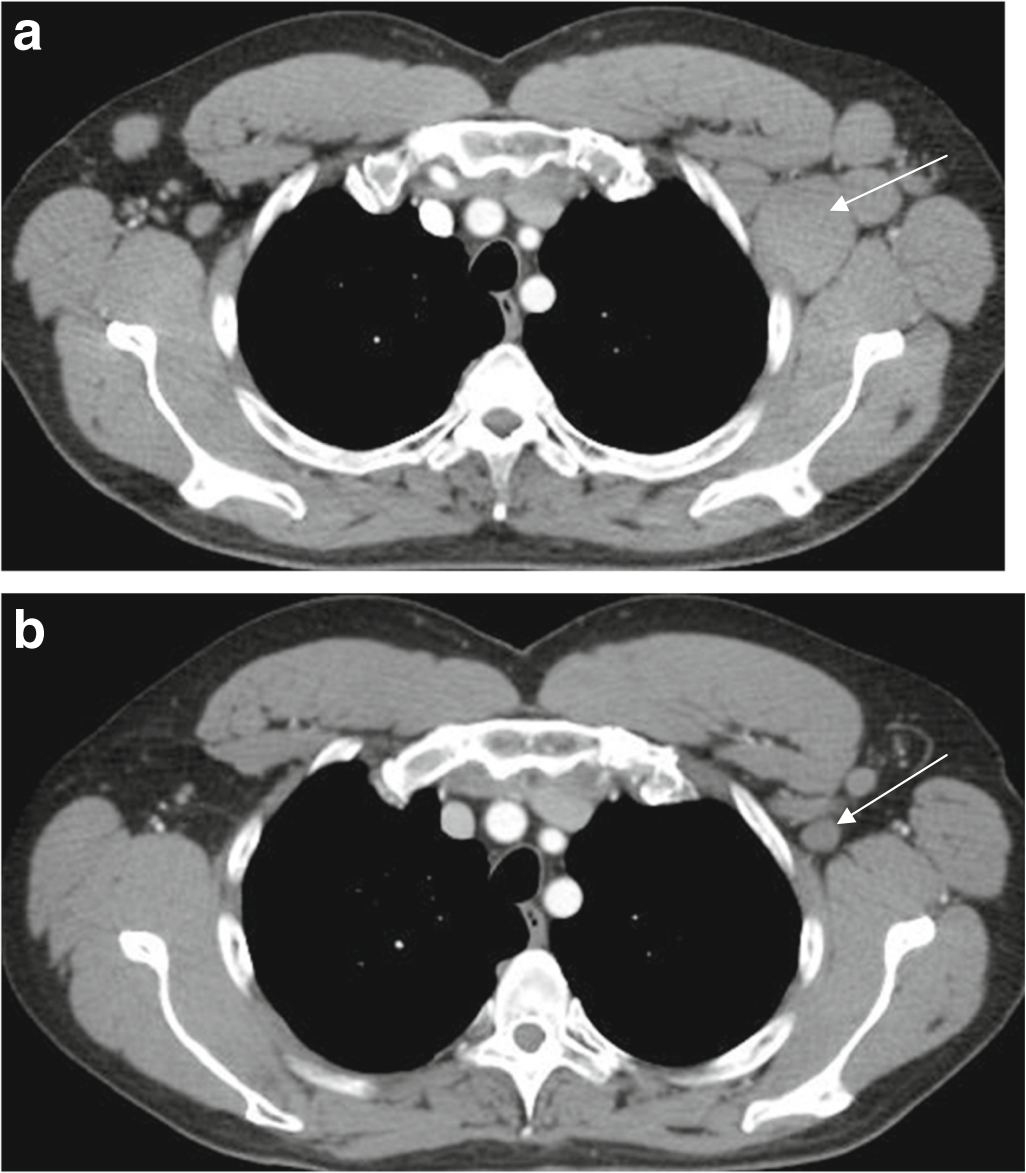
59-year-old male with follicular lymphoma was observed initially for 6 months after diagnosis but he progressed with increasing left axillary lymphadenopathy. **a** Axial CT images of the chest acquired during arterial phase reveal left axillary lymphadenopathy with the largest lymph node measuring 5 cm in short axis (white arrow). **b** Axial CT images of the chest acquired during arterial phase after 1 year on Ofatumumab demonstrate decrease in the size of left axillary lymph node which now measures less than 1 cm in short axis

Ofatumumab has been evaluated in clinical trials of elderly CLL patients. In a large phase III study comparing ofatumumab plus chlorambucil with chlorambucil alone, the overall response rate has higher in patients treated with combination therapy (82% versus 69%) and 14% demonstrated complete responses in the ofatumumab plus chlorambucil group [42]. In the patients receiving combination therapy, cytopenias, infections and infusion reactions were the most common toxicities; these adverse effects are similar in other ofatumumab studies. As seen with rituximab, pneumonitis is a rare potential toxicity associated with treatment (Fig. 6) [43, 44].

## Obinutuzumab (New anti-CD-20 antibody)

Obinutuzumab is a type II, glycoengineered antibody which has a slightly different orientation in binding to the CD20 receptor expressed on the B cell surface than rituximab, which produces greater apoptosis and more antibody mediated cytotoxicity [45]. It was FDA-approved in 2013 as a first line treatment (in combination with chlorambucil) in older CLL patients or in those with multiple co-morbidities.

**Fig. 7:**
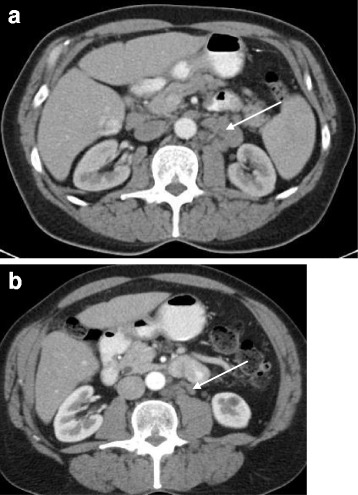
68-year-old male patient with relapsed CLL on Obinitizumab. **a** Axial CT images of the abdomen obtained in portal venous phase demonstrates prominent left para aortic lymph node measuring 3.4 × 1.6 cm (white arrow). **b** Axial CT images of the abdomen obtained 6 months after therapy demonstrate complete response with the lymph node measuring 1.7 × 0.9 cm (white arrow)

In a phase III study of 781 CLL patients treated with chlorambucil alone, rituximab plus chlorambucil, or obinutuzumab plus chlorambucil, obinutuzumab with chlorambucil was superior to the rituximab containing regimen, with prolongation of progression free survival and higher rate of complete response (20.7% versus 7%) (Fig. 7). Common adverse events associated with obinutuzumab include cytopenias and infusion reactions. Infections, especially pneumonia, have occurred in association with treatment. Similar to the previously cited adverse events associated with rituximab, reactivation of hepatitis B and progressive multifocal leukoencephalopathy have also been reported with obinutuzumab [46].

## Ibrutinib (Bruton Tyrosine Kinase Inhibitor)

Although antibody therapies have made an important contribution to the improvement in CLL therapy, small molecule targeted inhibitors such as ibrutinib have truly revolutionized the field in the last 5 years. One important difference between traditional chemoimmunotherapy and the newer targeted agents is that while the former are given as time-limited regimens (typically 6 months), the latter are typically given continuously until time of progression or unacceptable toxicity.

**Fig. 8:**
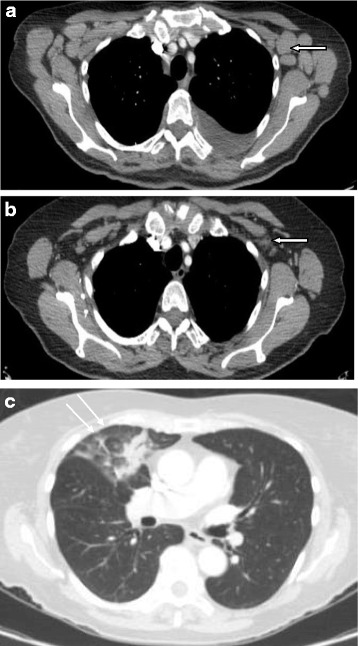
A 68-year-old female with CLL with 13q deletion on watchful waiting with progression of CLL **a** Axial CT of the chest obtained in arterial phase demonstrates bilateral axillary lymphadenopathy with the largest left axillary lymph node measuring 2 cm in short axis (white arrow). **b** Axial CT of the chest obtained 3 months after initiating Ibrutinib therapy reveals significant decrease in the lymphadenopathy with the axillary node now measuring less than a centimeter in short axis (white arrow). **c** Axial CT of the chest obtained in arterial phase as a part of restaging examination in the same patient reveals focal ground glass opacity in the right upper lobe (double arrows) raising concern for Ibrutinib pneumonitis

Ibrutinib is a small molecule which irreversibly inhibits the Bruton tyrosine kinase (BTK), by covalently binding to the cysteine-481 residueof BTK. It prevents downstream activation of transcription factors through blockade of the B cell receptor signaling pathway, which therefore inhibits CLL-cell survival and proliferation [47]. Ibrutinib is FDA-approved to treat any CLL patient in any line of therapy, including patients with del (17p) for whom it has become the standard of care for frontline therapy, and for those who have relapsed or progressed on prior treatments. A study by Burger et al. examined ibrutinib as a first line drug in CLL and compared to chlorambucil. In this report, 86% patients on ibrutinib monotherapy achieved an objective response compared to 35% on chlorambucil (Fig. 8) [48], and patients treated on the ibrutinib arm had a significant improvement in overall survival.

Ibrutinib is associated with a spectrum of adverse effects. For example, there is an associated 5–8% risk of atrial fibrillation, and increased risk of major and minor bleeding [49]. Ibrutinib should be held 3–7 days prior to and after any invasive procedure, due to the risk of periprocedural bleeding [50]. Other common adverse effects associated with ibrutinib include skin rash, diarrhea, hypertension and fatigue. Rarer complications include life-threatening central nervous system or gastrointestinal hemorrhage [51], as well as pneumonitis, which has been documented on chest CT, evidenced by diffuse bilateral ground glass opacities with or without consolidation (Fig. 8c) [52]. Ibrutinib is also associated with increased risk of infection with Pneumocystis jirovecii pneumonia and invasive aspergillosis [53]. Pneumocystis pneumonia commonly presents with interlobular septal thickening and diffuse ground glass opacities which are more predominant in the upper lobes [54]. Invasive pulmonary aspergillosis presents with nodules with central cavitation and aspergillosis of CNS presents as peripherally enhancing brain mass [55].

## Idelalisib (Delta-isoform phosphatidylinositol 3-kinaseinhibitor)

Idelalisib is a small molecule that selectively inhibits the delta isoform (δ) of phosphatidylinositol 3-kinase (PI3K), a form restricted primarily to leukocytes [56]. Inhibition of PI3Kδ inhibits the B cell receptor pathway, thereby inhibits B cell activation, proliferation and survival [56]. In 2014, the FDA approved idelalisib in combination with rituximab for relapsed or refractory CLL [2]. In a phase 2 study of idelalisib in 125 previously treated patients (median of four prior treatments) with indolent lymphoma (follicular, SLL, marginal zone lymphoma or lymphoplasmacytic lymphoma), 90% of the patients demonstrated decreased lymph node size, with 57% achieving complete or partial response [56], and the greatest efficacy in this study was seen in SLL patients.

**Fig. 9:**
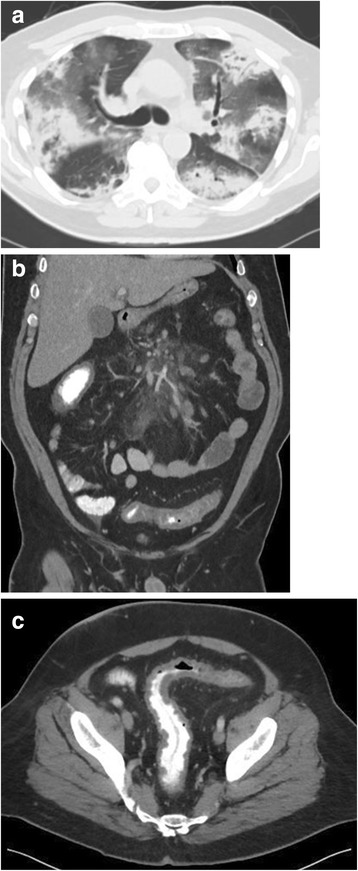
**a** 78-year-old female patient with CLL on treatment with Idelalisib with pneumonitis, axial CT of the chest obtained in arterial phase demonstrates bilateral patchy areas of ground glass and consolidative opacities. **b** and **c** 55-year-old male patient on Idelalisib therapy with colitis, reconstructed coronal CT images of the abdomen and pelvis in portal venous phase demonstrate short segment circumferential thickening of the colonic loop with mildly enlarged mesenteric lymph nodes and panniculitis. Axial CT images of the pelvis in portal venous phase show circumferential wall thickening of the sigmoid colon

Idelalisib is associated with several immune-mediated toxicities which are worthy of discussion. Common adverse events in the phase 2 study above include, but are not limited, to diarrhea, nausea, fatigue, cough, abdominal pain, pneumonia, elevated liver function tests, infection and cytopenias [56]. Idelalisib- related pneumonitis has been described in literature, and manifests as cough, dyspnea and fever at any time in the course of treatment. CT findings including diffuse bilateral ground glass opacities, consolidations, diffuse micronodules and pleural effusions (Fig. 9a) [57]. Idelalisib-associated colitis has been noted to typically occur after patients have been on drug for 6 to 9 months or later [58], and we have observed as case with CT findings of short segment colonic wall thickening, enlarged mesenteric lymph nodes and panniculitis (Fig. 9b and c). Another patient treated at our center with idelalisib was noted to have elevated liver function tests, and we observed imaging findings of decreased liver echogenicity and mild pericholecystic fluid on ultrasound. The multiple, potentially severe immune-mediated toxicities associated with this medication are delineated in a black box warning, for serious diarrhea, colitis, intestinal perforation, hepatotoxicity and pneumonitis [59]. Idelalisib is also associated with increased risk of opportunistic infections such as pneumocystis jirovecii [60].

## Venetoclax (B-cell leukemia/lymphoma-2 inhibitor)

Venetoclax binds to anti-apoptotic B-cell leukemia/lymphoma-2 protein (Bcl-2) and displaces pro-apoptotic BH3-only proteins, which then induce rapid apoptosis in CLL cells [61]. Venetoclax was FDA-approved in 2016, to treat CLL with 17p deletion, refractory to treatment with at least one prior regimen [2]. In the phase 1 first in human studyin patients with relapsed or refractory CLL, most of whom had received multiple prior therapies and many of whom had del (17p), 116 patients received venetoclax. An overall response rate of 79% was reported, with complete response in 20% of the patients [61]. Tumor lysis syndrome (TLS) was noted in several patients and in some cases was severe, indicating the power of this drug but also requiring careful risk stratification, prophylaxis, and management of TLS. Other adverse associated events in the study patients included diarrhea, upper respiratory tract infection and nausea. Grade 3/4 neutropenia occurred in about 40% of patients, but the rate of febrile neutropenia was low. The findings from this phase I study were subsequently confirmed in a landmark study of 107 CLL patients with relapsed or refractory del (17p) disease, and the response rates in this high risk population were similar to those seen in the broader population in the phase I study [62].

## Conclusion

The last 5 years have witnessed an exciting evolution of targeted therapy in CLL/SLL. Relatively few radiology- based papers have been written on this expansive subject; rather, case series have reported on the various drug-associated toxicities attributed to specific agents. Considering the logical development of the targeted agents and class-specific mechanisms of action, it becomes easier to appreciate the class-specific toxicities related to the drugs.

In general, indolent lymphoma patients on treatment are at risk of infectious complications. More specifically, the anti-CD20 antibodies rituximab, ofatumumab and obinutuzumab have been associated with reactivation of hepatitis B and rarely, progressive multifocal leukoencephalopathy. Pneumonitis has also been noted with these antibodies. The anti-CD52 antibody alemtuzumab has been associated with various infectious complications, especially CMV. Small molecule inhibitors have distinct adverse event profiles, including bleeding, atrial fibrillation and diarrhea with ibrutinib, potentially severe colitis, hepatotoxicity and pneumonitis with idelalisib, and tumor lysis syndrome, neutropenia, and diarrhea with venetoclax. Despite these potential toxicities, each of these agents is well-tolerated for the majority of patients and each has demonstrated dramatically improved response rates and progression free survival compared to chemoimmunotherapy in heavily-pretreated patients, leading to substantial improvements in overall survival, particularly for those patients with high risk del (17p) CLL. Ongoing studies combining these novel agents hold promise to further revolutionize the treatment of CLL and hold the potential to even further reduce or possibly even eliminate the need for chemoimmunotherapy in many patients. It is worth noting that although these new drugs also have activity in other low grade B cell non-Hodgkin lymphomas, their efficacy is generally lower than in CLL, and as such it is likely that these conditions will also benefit from combination therapeutic strategies, which is already being explored in the clinic.

Given the indolent nature of CLL and other low grade lymphomas, the spectrum of available treatments today and the associated prolonged survival of affected patients, it is likely that radiologists will encounter more imaging studies of patients treated with these novel drugs. It is necessary to be familiar with the expected response patterns and common toxicities associated with targeted agents to optimize care of this growing population.

## Abbreviations

ARDS: Acute respiratory distress syndrome
Bcl2: B-cell leukemia/lymphoma 2
BCR: B-cell receptor
BR: Bendamustine, rituximab
BTK: Bruton tyrosine kinase
CECT: Contrast-enhanced CT
CLL: Chronic lymphocytic leukemia
CMV: Cytomegalovirus
CNS: Central nervous system
CR: Complete response
FCR: Foscarnet, cyclophosphamide, rituximab
FDA: Food and Drug Administration
FISH: Florescence in-situ hybridization
HRCT: High resolution CT
IGHV: Immunoglobulin heavy chain variable
iwCLL: International Workshop on Chronic Lymphocytic Leukemia
LDH: Lactate dehydrogenase
MRD: Minimal residual disease
PI3K: Phosphotidylionositol 3 kinase
PR: Partial response
PR-L: Partial response with lymphocytosis
RCVP: Rituximab, cyclophosphamide, vincristine, prednisone
SLL: Small lymphocytic leukemia
TLS: Tumor lysis syndrome
ZAP-70: Zeta-associated protein 70
